# Lung density change after SABR: A comparative study between tri-Co-60 magnetic resonance-guided system and linear accelerator

**DOI:** 10.1371/journal.pone.0195196

**Published:** 2018-04-02

**Authors:** Eunji Kim, Hong-Gyun Wu, Jong Min Park, Jung-in Kim, Hak Jae Kim, Hyun-Cheol Kang

**Affiliations:** 1 Department of Radiation Oncology, Seoul National University College of Medicine, Seoul, Republic of Korea; 2 Cancer Research Institution, Seoul National University College of Medicine, Seoul, Republic of Korea; 3 Institute of Radiation Medicine, Medical Research Center, Seoul National University, Seoul, Republic of Korea; University of Sydney, AUSTRALIA

## Abstract

Radiation-induced lung damage is an important treatment-related toxicity after lung stereotactic ablative radiotherapy (SABR). After implementing a tri-^60^Co magnetic-resonance image guided system, ViewRay^TM^, we compared the associated early radiological lung density changes to those associated with a linear accelerator (LINAC). Eight patients treated with the tri-^60^Co system were matched 1:1 with patients treated with LINAC. Prescription doses were 52 Gy or 60 Gy in four fractions, and lung dose-volumetric parameters were calculated from each planning system. The first two follow-up computed tomography (CT) were co-registered with the planning CT through deformable registration software, and lung density was measured by isodose levels. Tumor size was matched between the two groups, but the planning target volume of LINAC was larger than that of the tri-^60^Co system (*p* = 0.036). With regard to clinically relevant dose-volumetric parameters in the lungs, the ipsilateral lung mean dose, *V*_10Gy_ and *V*_20Gy_ were significantly poorer in tri-^60^Co plans compared to LINAC plans (*p* = 0.012, 0.036, and 0.017, respectively). Increased lung density was not observed in the first follow-up scan compared to the planning scan. A significant change of lung density was shown in the second follow-up scan and there was no meaningful difference between the tri-^60^Co system and LINAC for all dose regions. In addition, no patient developed clinical radiation pneumonitis until the second follow-up scan. Therefore, there was no significant difference in the early radiological lung damage between the tri-^60^Co system and LINAC for lung SABR despite of the inferior plan quality of the tri-^60^Co system compared to that of LINAC. Further studies with a longer follow-up period are needed to confirm our findings.

## Introduction

Stereotactic ablative radiotherapy (SABR) is an established treatment option for early stage non-small-cell lung cancer (NSCLC) and pulmonary oligometastases [1, 2]. It has been reported that lung SABR is generally well tolerated, with low post-treatment mortality [3]. However, the risk of radiation-induced lung damage (RILD) that is not an uncommon complication after conventional radiotherapy needs to be considered when treating a patient with poor lung function or underlying lung disease, as the difference in treatment-related morbidity and mortality could be substantial rather than generally expected [4, 5]. Moreover, if the respiratory movement of tumor is considerable, especially for tumors in the lower lobe, the risk of RILD becomes higher with the increased planning target volume (PTV) when compensating for tumor movement by defining the internal target volume (ITV).

Lung density is regarded as an objective predictive parameter for clinical RILD. Objective methods for assessing RILD after radiotherapy may be useful to detect the lung injury and to improve the quality of radiation. The quantitative measurement of RILD based on CT scan has been reported in several studies [6–11]. Previous study demonstrated a dose-response relationship for quantitative lung density changes after SABR. The radiation dose threshold for density increase was 6 Gy and a prominent increase was observed in the region receiving > 20 Gy [7].

MRIdian^TM^ (ViewRay Inc., Cleveland, OH) is a tri-^60^Co magnetic resonance image (MRI)-guided radiation therapy system and provides a respiratory gating technique without an external surrogate [12, 13]. Moreover, it has real-time cine images to monitor internal organ movement, thus the target margin can be reduced from the ITV-based approach, and more importantly it could be the most reliable treatment option for patients who have unpredictable breathing patterns. The quality of the tri-^60^Co system for lung SABR has previously been shown to be inferior to that of the ITV-based linear accelerator (LINAC) plan when the PTV volume is less than 10 cc [14]. The multileaf collimator (MLC) leaf width of the tri-^60^Co system is larger than that of LINAC with the Millennium 120^TM^ or high-definition 120^TM^ MLC (Varian Medical Systems, Palo Alto, CA), which could deteriorate the plan quality. In addition, cobalt sources result in the relatively large penumbra and low penetrability, which could also degrade the plan quality. Because radiotherapy using tri-^60^Co system has both advantages and disadvantages compared with LINAC as described above, it is unclear whether there is any difference in treatment outcomes depending on the treatment modalities (tri-^60^Co system vs. LINAC). Therefore, this study was intended to investigate the difference in RILD between tri-^60^Co system and LINAC. We compared changes in lung density for tri-^60^Co system versus LINAC-based SABR to provide quantitative parameters that could serve as an early detection of RILD.

## Materials and methods

### Patient selection

This study was done after the approval of institutional review board from Seoul National University Hospital (No. H-1704-013-842). The informed consent was waived, because the data were fully anonymized before we access to them. We retrospectively reviewed patients who had received lung SABR at our institution from 2015 to 2016. They were excluded if the patient had previously received radiotherapy in their thorax or showed locoregional recurrence during the follow-up period. The toxicity of SABR using the tri-^60^Co system was compared with that of LINAC-based SABR, by matching patients at a 1:1 ratio. Matches were made in the following order of importance: dose/fractionation, tumor size, tumor location, and age. The size of the tumor was measured by trained radiologists using diagnostic chest computed tomography (CT).

### Treatment details

For LINAC SABR, patients underwent ten phase four-dimensional CT scans (Brilliance CT bigbore^TM^, Philips, Cleveland, OH) with 2 mm slice thickness. The gross tumor volume (GTV) was delineated in 10 phases, and physicians confirmed that the ITV delineated on maximum intensity projection images included GTVs on all phases. PTV margin was usually 5 mm, but it was 3 mm for two patients with highly reproducible breathing pattern. The dose distributions were calculated with the analytic anisotropic algorithm in the Eclipse^TM^ system (Varian Medical Systems, Palo Alto, CA). The volumetric modulated arc therapy (VMAT) plans were delivered with a 6 MV flattening filter-free beam of Truebeam STx^TM^ (Varian Medical Systems, Palo Alto, CA) after imaging with a kV cone-beam CT.

For SABR with the tri-^60^Co system, patients underwent a planning MRI and three-dimensional CT scan with quiet breathing. GTV was delineated on one-phase MR images. PTV was defined with a margin of 3 mm from the GTV. Exceptional margin of 5 mm was applied in two patients who were expected to have prolonged treatment time with gating technique. Static intensity-modulated radiation therapy (IMRT) plans were generated with MRIdian^TM^ system (ViewRay Inc., Cleveland, OH), and dose distributions were calculated with the Monte Carlo calculation algorithm. The treatment planning system delivered the SABR plan using one to three cobalt-60 sources at a time. The number of beams used for each plan was from 5 to 14, and the number of gantry positions was from 5 to 7. We treated patients with a respiratory gating technique based on real-time MR images provided by the tri-^60^Co system.

Dose-fractionation schedules were determined individually based on tumor size, location, and organs at risk. Patients were treated with either 52 or 60 Gy in four fractions. Routine 3D chest CT scans at inspiratory breath hold were performed at one and 4–6 months post-treatment. We defined the normal lung as bilateral lungs excluding the PTV because of the use of different treatment planning systems and the variation of GTVs delineated on MRI and CT. For the normal lung, clinically relevant dose-volumetric parameters (*V*_5Gy_, *V*_10Gy_, *V*_20Gy_, *D*_1000cc_, and *D*_1500cc)_ were calculated in each planning system.

### Image registration and analysis

**Fig 1:**
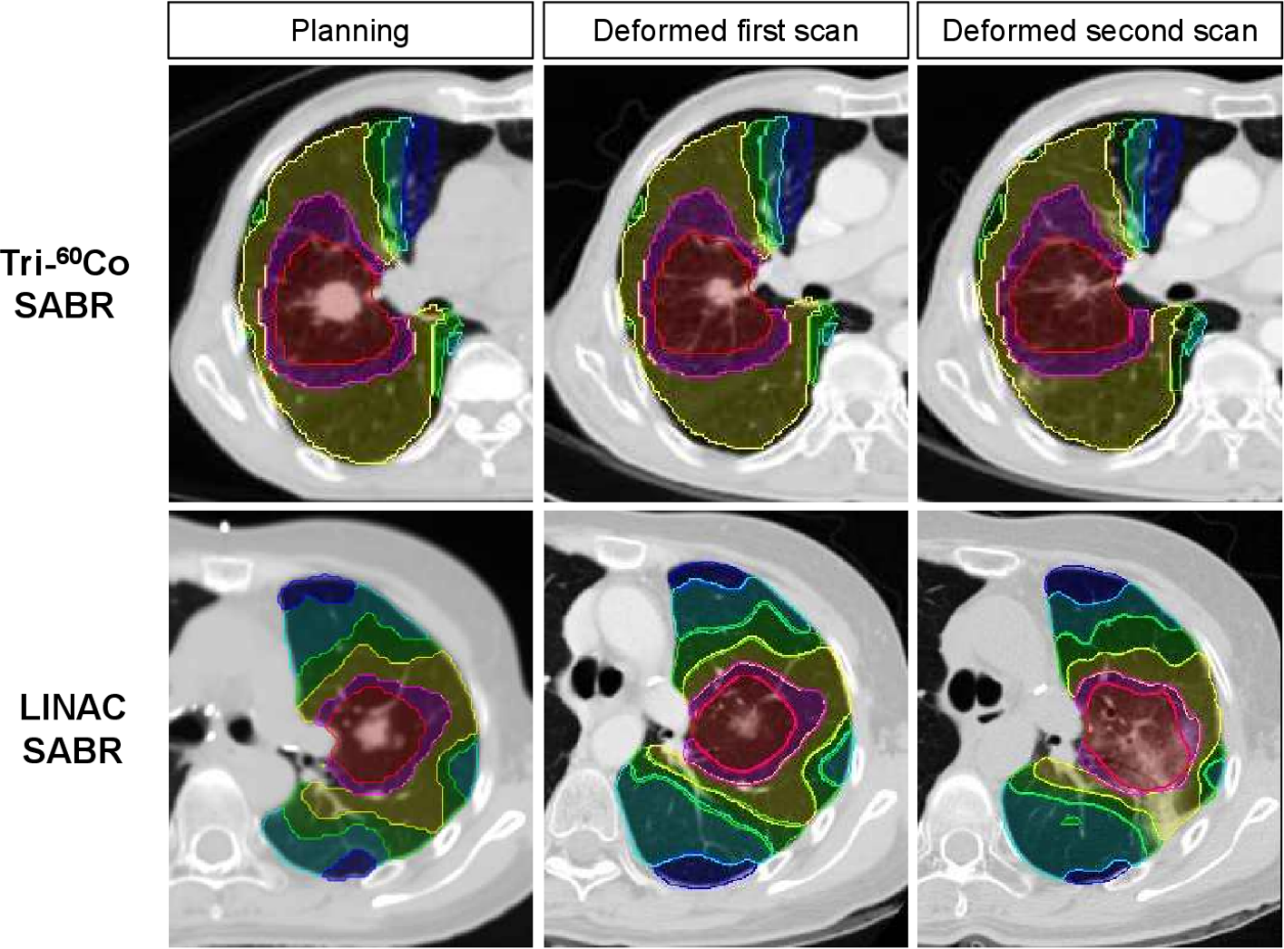
The representative deformable image registration before and after radiotherapy overlaid with isodose distributions. Isodose areas shown are as follows: dark blue (6–12 Gy), cyan (12–18 Gy), green (18–24 Gy), yellow (24–36 Gy), magenta (36–48 Gy), and red (> 48 Gy).

Contours of lung, GTV, and isodose levels were exported from the planning system. We chose the isodose levels as follows in a previously reported study: 0.5 Gy, 3 Gy, 6 Gy, 12 Gy, 18 Gy, 24 Gy, 36 Gy, and 48 Gy [7]. Post-treatment CT scans were overlaid on a planning CT scan at end-inspiration phase using deformable registration with MIM^TM^ version 5.4 (Fig 1, MIM Software Inc., Cleveland, OH), and changes in the lung density (measured in Hounsfield unit, HU) were assessed. The CT density of contralateral lung irradiated at < 3 Gy was analyzed to calibrate the baseline of serial CT scans. Wilcoxon signed-rank test and McNemar’s test were performed with SPSS version 23.0 (SPSS Inc., Chicago, IL), and two-sided *p*-values of < 0.05 were considered statistically significant.

The primary outcome of this study is paired difference between lung density changes in tri-^60^Co SABR and those in LINAC SABR. Since the standard deviation of difference in lung density changes has not been established, we assumed it from the observed outcome of the current study. Dispersion of paired differences of lung density changes were varied according to radiation dose in the second follow-up scan. The smallest standard deviation of paired differences of lung density changes was approximately 36 HU at 0.5–3 Gy and the largest one was 220 HU at above 48 Gy. When all the dose fractions were pooled, standard deviation of paired differences of lung density changes was approximately 100 HU. Therefore, we estimated the minimum detectable difference in lung density changes assuming the difference in lung density changes follows normal distribution and their standard deviation of 100. The size of 8 pairs achieves 80% power to detect a difference of 115.6 between the null hypothesis mean of 0.0 and the alternative hypothesis mean of 115.6 with an estimated standard deviation of 100.0 and with a significance level of 0.05 using a two-sided one-sample t-test (paired t-test) [15].

## Results

### Patient and treatment characteristics

In total, 16 patients treated with lung SABR using the tri-^60^Co system or LINAC were eligible for this study. Patients with early lung cancer (n = 13) or metastatic lesions (n = 3) were treated, and there was no central lesion. All patients underwent routine follow-up chest CT scans. The median intervals for first and second follow-up CT scans from the end date of radiotherapy were 5.5 weeks (range: 4–7 weeks) and 20.5 weeks (range: 16–31 weeks), respectively. No symptoms consistent with radiation pneumonitis were reported in the treated patients. The patients and treatment characteristics are shown in Table 1. Patient matching was well performed based on tumor size and location, dose/fractionation, and age; although there was no significant difference in tumor size between the two groups (*p* = 0.291), PTV showed significant difference according to treatment modalities (*p* = 0.036). The clinically relevant dosimetric parameters in lung are shown in Table 2. The mean doses to both lungs of tri-^60^Co SABR plans were higher than those of the LINAC SABR plans with statistical significance (mean dose to the ipsilateral lung, 7.2 Gy for tri-^60^Co plans vs. 4.7 Gy for LINAC plans, *p* = 0.012; mean doses to the contralateral lung, 1.4 Gy for tri-^60^Co plans vs. 0.7 Gy for LINAC plans, *p* = 0.036). The volumes of normal lung receiving 10 Gy and 20 Gy were statistically larger for tri-^60^Co SABR plans (*p* = 0.036 and *p* = 0.017, respectively). The other parameters such as *D*_1000cc_, and *D*_1500cc_ were poor in tri-^60^Co plans without statistical significance (*p* = 0.069 and *p* = 0.071, respectively).

**Table 1:** Patient and treatment characteristics.

Variable	Tri-^60^Co SABR (n = 8)	LINAC SABR (n = 8)	*p*-value
Age (years)	73 ± 7^a^	71 ± 9^a^	0.779*
Gender			
Male	4	6	0.625^†^
Female	4	2	
Tumor size (cm)	1.44 ± 0.58^a^	1.69 ± 0.42^a^	0.291*
Planning target volume (cc)	9.06 ± 7.02^a^	14.78 ± 3.97^a^	0.036*
Diagnosis			
Primary lung cancer	7	6	1.0^†^
Metastatic lung cancer	1	2	
Tumor location			
Upper lobe	5	2	0.375^†^
Lower lobe	3	6	
Dose/fraction			
60 Gy/ 4 fx	7	7	1.0^†^
52 Gy/ 4 fx	1	1	

^a^Values are presented as mean ± standard deviation.

*Wilcoxon signed-rank test.

^†^McNemar’s test.

**Table 2:** Dose-volumetric parameters in lung.

Variable	Tri-^60^Co SABR (n = 8)	LINAC SABR (n = 8)	*p*-value*
Ipsilateral lung mean dose (Gy)	7.17 ± 1.55	4.66 ± 2.42	0.012
Contralateral lung mean dose (Gy)	1.35 ± 0.6	0.67 ± 0.35	0.036
*V*_5Gy_ (cc)	603.41 ± 280.21	313.02 ± 158.21	0.050
*V*_10Gy_ (cc)	396.62 ± 201.28	186.42 ± 83.23	0.036
*V*_20Gy_ (cc)	218.36 ± 153.51	92.09 ± 40.43	0.017
*D*_1000cc_ (Gy)	2.07 ± 1.92	0.89 ± 0.64	0.069
*D*_1500cc_ (Gy)	0.94 ± 0.9	0.37 ± 0.24	0.071

*V*_nGy_ = total normal lung volume receiving n Gy; *D*_ncc_ = dose received by at least n volume of a total normal lung.

Values are presented as mean ± standard deviation.

*Wilcoxon signed-rank test.

### Changes in CT density (HU)

**Fig 2:**
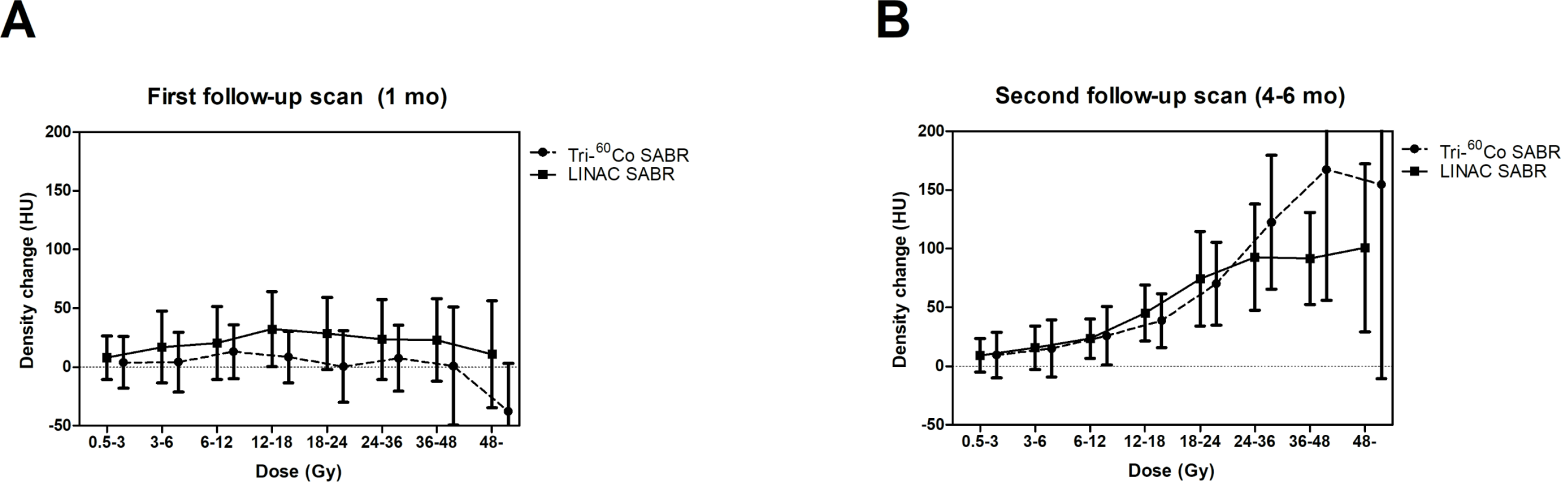
Mean lung density changes according to the treatment modality at (A) first follow-up scan and (B) second follow-up scan. Error-bars represent 95% confidence interval.

The changes in lung density in the first follow-up scan are as shown in Fig 2A. There was no correlation between radiation dose and density change in the first follow up scan. The mean lung density changes in the area above 48 Gy were -37.79 HU [95% confidence interval (CI), -78.38:2.8] in tri-^60^Co SABR and 10.98 HU (95% CI, -34.65:56.61) in LINAC SABR. Although the mean lung density changes of the tri-^60^Co system were less in all dose regions of the first post-treatment CT, there were no significant differences between tri-^60^Co SABR and LINAC SABR (all with *p* > 0.05).

In the second follow-up CT scan, the density of lung irradiated above 6 Gy was significantly increased after SABR compared to the baseline (Fig 2B). For tri-^60^Co SABR, the mean lung density changes (HU) in 6–12 Gy, 12–18 Gy, 18–24 Gy, 24–36 Gy, 36–48 Gy, and > 48 Gy were 25.6, 38.5, 69.9, 122.4, 167.1, and 154.2, respectively (*p* = 0.036, 0.012, 0.012, 0.012, 0.012, and 0.025, respectively). For patients treated with LINAC SABR, the mean lung density changes (HU) in 6–12 Gy, 12–18 Gy, 18–24 Gy, 24–36 Gy, 36–48 Gy, and > 48 Gy were 23.6, 45.4, 74.5, 92.7, 91.8, and 100.8, respectively (*p* = 0.013, 0.003, 0.003, 0.002, 0.001, and 0.012, respectively). However, there were no significant differences in lung density changes according to treatment modality (tri-^60^Co system vs. LINAC) for all dose regions (0.5–3 Gy, *p* = 0.859; 3–6 Gy, *p* = 0.961; 6–12 Gy, *p* = 0.871; 12–18 Gy, *p* = 0.999; 18–24 Gy, *p* = 0.982; 24–36 Gy, *p* = 0.978; 36–48 Gy, *p* = 0.545; > 48 Gy, *p* = 0.665). Furthermore, lung density changes were not associated with age (< 75 years vs. ≥ 75 years, *p* = 0.382), gender (male vs. female, *p* = 0.571), smoking history (ex-/current smoker vs. never-smoker, *p* = 0.125), underlying lung disease (yes vs. no, *p* = 0.559), GOLD score (0–1 vs. 2–3, *p* = 0.461), PTV (≤ 14 cc vs. > 14 cc, *p* = 0.345), or tumor location (upper vs. lower lobe, *p* = 0.143).

## Discussion

This study demonstrates lung density changes after SABR in patients treated with two comparable treatment systems, the tri-^60^Co MRI-guided system and LINAC. The shown dosimetric parameters were worse in the tri-^60^Co system. However, both treatment systems kept the normal organ dose constraint suggested in the Radiation Therapy Oncology Group (RTOG), and there was no significant difference in lung density changes between two treatment systems. The RTOG conducted the clinical studies (RTOG 0813 and 0915) to clarify the tolerance of critical organs during the lung SABR. In our study, *D*_max_ for spinal cord, esophagus, and heart were 14 Gy, 18 Gy, and 12 Gy, respectively, and those were under the dose constraints the RTOG proposed [16–18].

RILD including radiation pneumonitis and fibrosis is one of the toxicities of most concern in thoracic radiation therapy. The incidence of moderate-to-severe radiation pneumonitis was reported as approximately 10–20% in conventionally fractionated radiotherapy (1.8–2 Gy per fraction), and an increased risk of toxicity was expected for SABR due to the large fraction size [19–20]. However, treatment-related grade 3–4 pulmonary toxicity was 16% in the multicenter study for patients treated with SABR [1]. In a pooled analyses of two randomized trials, two patients (6%) treated with SABR showed dyspnea or cough with a median follow-up of 40.2 months [21]. Furthermore, retrospective studies reported that the incidence of radiation pneumonitis above grade 2 was in the range 6–21% and pulmonary function test changes were minimal after lung SABR [22–24]. Among the patients treated with tri-^60^Co lung SABR in our institution, none have ever complained of clinical symptoms of radiation pneumonitis. To the best of our knowledge, this is the first study describing treatment toxicity due to tri-^60^Co lung SABR.

Patient-related factors such as age, smoking history, tumor size, tumor location, performance score, and gender- and treatment-related factors including chemotherapy regimen and lung dosimetry have been suggested as predictors for the development of RILD [25–27]. In regards to radiographic change, Kishan et al. [28] reported that age, years since quitting smoking, and GOLD (Global Initiative for Chronic Obstructive Lung Disease) score were significantly associated with volumes of radiographic fibrosis following SABR. They found the predominant patterns of fibrosis peaking volumes of fibrosis at 6 and 12 months. In our study, the clinical factors including underlying lung disease, smoking history, and GOLD score were not associated with lung density changes. Because our patients have a short follow-up period compared to the previous study, additional observations are needed to confirm the relationship between clinical factors and radiologic changes.

Evaluating the CT density changes has been considered as an objective and quantitative method to assess lung toxicity after radiotherapy [6–11]. Palma et al. [7] showed a dose-response relationship for lung density changes and the increase of 100 HU in the areas above 40 Gy when the PTV size was less than 50 cc at post-SABR 6–9 months. However, the optimal cut-off value of the density change (HU) with clinical significance has not been proposed yet. This is probably due to the low toxicity rate of lung SABR, and our study was difficult to assess because there was no lung toxicity after SABR. Further studies can help predict the therapeutic toxicity if one knows about the appropriate cut off value of the CT density change.

In general, radiological changes occur at least three months and stabilize 9–12 months after conventional radiotherapy [29–33]. Takeda et al. [34] reported that radiation injury may occur during the first year after SABR. Phernambucq et al. [12] performed follow-up CT scans up to two years after treatment. Radiological lung density increased from 2.5 months after concurrent thoracic chemoradiotherapy, but stabilized at one year. However, it was reported that radiation fibrosis may develop and continue more than one year after SABR [20, 35]. In our study, there was no significant increase in lung density for the first follow-up scan at 5.5 weeks, but an increase in lung density was evident in the second follow-up scan taken at 20.5 weeks.

Internal organ motion was not considered when generating the PTV for the tri-^60^Co system, because it has a great advantage of being able to monitor the movement of the tumor through MR images during treatment. In this study, tumor size was matched between patients treated with the tri-^60^Co system and those treated with LINAC, but the target volume of the tri-^60^Co system was smaller than that of LINAC. Palma et al. [7] demonstrated that greater caution is warranted in delivering radiation to patients with large tumors and poor lung function. CT density changes were associated with PTV in patients treated with LINAC SABR, and the increase in CT density was greater in patients with PTV > 100 cc at a low dose area. Unlike in the previous study, we could not find any correlation with lung density changes and PTV, presumably because the PTV of the patients included in our study was small compared to that of the other study. Therefore, the tri-^60^Co system should be considered in the treatment of large tumor with ITV-based LINAC SABR.

MRIdian^TM^ uses cobalt-60 radiation beams to avoid interference with the MR unit [13]. Park et al. [14] compared the IMRT plans of tri-^60^Co SABR and VMAT plans of LINAC SABR for lung cancers located in the lower lobe. They created the PTV for LINAC plans from the ITV and the PTV for the tri-^60^Co plans from the GTV, which is the same as in our study and they analyzed the qualities of the two plans for the same patient. Although there was no significant difference between tri-^60^Co and LINAC plans in most dose-volumetric parameters for organs at risk (e.g. spinal cord, esophagus, heart, bronchi, rib, and skin), tri-^60^Co plans were always significantly worse than LINAC plans for the values of a normal lung. Furthermore, the target conformity index and the homogeneity index were poor in the tri-^60^Co system compared to those of LINAC. Park et al. [14] suggested that the poor plan quality of the tri-^60^Co system might be due to the large MLC width and the low penetrating power of cobalt sources.

Wooten et al. [36] reported the performance of the treatment planning and verified the delivery of IMRT with the tri-^60^Co system according to the American Association of Physicists in Medicine (AAPM) Task Group 119. They also performed a comparative planning study between tri-^60^Co IMRT plans and LINAC IMRT plans on various diseases [37]. Tri-^60^Co plans achieved similar PTV coverages and tolerable doses of normal organs. Although mean doses of organs at risk of LINAC plan were lower compared with the tri-^60^Co system in the low dose area (< 20 Gy), there was comparable sparing for normal organs with mean doses > 20Gy. There were limited number of fractionated IMRT plans (6 cases), and the target volumes of both tri-^60^Co plans and LINAC were the same as in the previous study, which could not maximize the advantage of the gating function based on the real-time MR images of the tri-60 cobalt system. When we analyzed dose-volumetric parameters of normal lung in this study, the values of lung mean doses, *V*_5Gy_ and *V*_10Gy_, were significantly higher in the tri-^60^Co system than in LINAC. However, it did not lead to difference in lung density changes between the tri-^60^Co system and LINAC and no clinical pneumonitis was observed in either group.

The current study had several limitations. First, although possible confounders were adjusted by using 1:1 patient matching, unknown factors may still remain. Second, due to incomplete pairing, the detectable effect size comparing the density changes from paired t-test could be overestimated. However, due to the rarity of studies demonstrating the toxicities after lung SABR with the tri-^60^Co system, this study may provide valued data regarding the tri-^60^Co system which treats patients with smaller PTV and monitors the tumor in real time.

## Conclusions

In conclusion, the difference in early lung density changes between tri-^60^Co system SABR and LINAC SABR did not reach statistical significance. Although the lung dosimetric parameters of tri-^60^Co plans were poor compared to those of the LINAC plans, our results suggest that tri-^60^Co SABR could be performed safely. Moreover, the advantage of tri-^60^Co system’s ability to monitor tumor movement can reduce the planning target volume and it seems important to patients with limited lung function. However, further follow-up and more experience are needed to assess late lung damage.
