# Correction: Lung density change after SABR: A comparative study between tri-Co-60 magnetic resonance-guided system and linear accelerator

**DOI:** 10.1371/journal.pone.0197799

**Published:** 2018-05-17

**Authors:** Eunji Kim, Hong-Gyun Wu, Jong Min Park, Jung-in Kim, Hak Jae Kim, Hyun-Cheol Kang

The following information is missing from the Funding section: This study was also supported by the National Research Foundation of Korea (NRF), which is funded by the Korea government (MEST, Grant No. 2014M2A2A7055063).
